# Is Guillain–Barré syndrome related to systemic lupus erythematosus or other autoimmune diseases?

**DOI:** 10.3389/fneur.2023.1336794

**Published:** 2024-01-10

**Authors:** Linpei Jia, Fengming Ni, Hong-Liang Zhang

**Affiliations:** ^1^Department of Nephrology, Xuanwu Hospital, Capital Medical University, Beijing, China; ^2^Department of Gastroenterology, Digestive Endoscopy Center, First Hospital, Jilin University, Changchun, China; ^3^Department of Life Sciences, National Natural Science Foundation of China, Beijing, China

**Keywords:** Guillain-Barré syndrome, systemic lupus erythematosus, peripheral nervous system, cerebrospinal fluid, autoimmune disease

## Introduction

Guillain–Barré syndrome (GBS) is a group of acute immune-mediated disorders in the peripheral nervous system (PNS). Acute-onset, rapidly progressive, symmetric weakness of the limbs along with hyporeflexia or areflexia is the typical feature of GBS (1). GBS is associated with both infections and non-infectious factors, which may trigger autoimmune responses (2). Bacterial and viral infections are common infectious triggers. The onset of GBS is typically preceded by upper respiratory or gastrointestinal infections prior to disease onset, implying that the host's response to infections contributes to disease pathogenesis. Approximately two-thirds of cases are preceded by infections, usually within 4–8 weeks before disease onset. *Campylobacter jejuni* is the most frequently identified infectious agent associated with subsequent development of GBS, accounting for up to 30% of infections (3), whereas cytomegalovirus has been identified in up to 10% of cases (4). Coronavirus disease 2019 (COVID-19) and its vaccines have been associated with GBS. Acute inflammatory demyelinating polyneuropathy (AIDP) is the prototype of GBS, and variants of GBS include generalized axonal subtypes, i.e., acute motor axonal neuropathy (AMAN) and acute motor and sensory axonal neuropathy (AMSAN) (5). Regional variants such as Miller Fisher syndrome (MFS) and the pharyngeal-cervical-brachial variant (PCB) are relatively rare. Nerve conduction studies (NCSs) and antibody detection can help discriminate different subtypes of GBS. Albuminocytologic dissociation, i.e., increased levels of albumin with a normal cell count in the cerebrospinal fluid (CSF) is a hallmark of GBS and can be detected in up to 90% of patients if lumbar puncture is performed and CSF is examined 3 weeks after the onset of disease (5). Immunomodulatory therapies, including intravenous immunoglobulins (IVIg) and plasma exchange (PE), have long been the first-line treatment for GBS.

## Pathogenesis of GBS and its implications

As the most common cause of acquired flaccid paralysis worldwide (2), GBS results from a synergistic interaction of cellular and humoral immune responses to autoantigens in the PNS (6). In *C. jejuni*-related GBS, there is good evidence to support an autoantibody-mediated immune process against shared structural components of peripheral nerves and the microorganism, which is termed molecular mimicry (7). Unlike chronic inflammatory demyelinating polyradiculoneuropathy (CIDP), a chronic disease in the PNS, GBS is not typically associated with any autoimmune disease. Even more importantly, corticosteroids administered alone do not hasten recovery from GBS or affect the prognosis (8). In a recent systematic review, the authors used the term “SLE-related GBS” to describe polyneuropathy attributable to SLE and “pure GBS” to describe GBS attributable to other conventional triggers, such as infections (9). The definition of SLE-related GBS in this case series, even if not totally, is ambiguous and somewhat misleading, as in another case report, the patient responded poorly to IVIg but well to intravenous methylprednisolone and cyclophosphamide (10).

## Discussion

GBS is not considered to be related to any chronic autoimmune disease for several reasons. Unlike many autoimmune disorders, GBS is more common in males than females and usually presents with a monophasic course (11). The only two proven therapies for GBS are IVIg and PE and they appeared to be the same effective in randomized controlled trials. More importantly, no evidence supports the efficacy of immunosuppressants or immunomodulators, which are widely used to treat various autoimmune diseases. Instead, GBS has been associated with immune checkpoint inhibitors (ICIs) and TNF-alpha antagonists (12). As such, the differential diagnosis of GBS includes autoimmune disease-associated polyneuropathy, e.g., SLE-associated polyneuropathy.

Polyneuropathy refers to a disease entity in which structural or functional impairment of the PNS is involved, with various etiologies, including infection, systemic or metabolic disorders, autoimmune disorders, e.g., SLE, rheumatoid arthritis, and Sjögren's syndrome, exposure to toxic compounds and medicinal therapies. As an autoimmune disease involving multiple organs, SLE is generally treated by immunosuppressive agents. Polyneuropathy is relatively common in SLE, with a prevalence ranging from 1.5 to 36%, and occurs more frequently in patients with involvement of the central nervous system (CNS) and high disease activity (13). The involvement of the PNS is usually insidious and slowly progressive in patients with SLE. SLE-associated polyneuropathy may show autoantibody levels correlated with SLE disease activity, and these patients may benefit from immunosuppressive therapies. However, patients with classical GBS usually present with rapidly progressive symmetric weakness with or without sensory loss. As such, the diagnosis of GBS in patients with SLE and peripheral nerve involvement needs to be meticulous.

The onset of GBS-like syndrome does not preclude the prior involvement of the peripheral nerves and an acute aggravation due to various reasons, e.g., infection, or medication toxicity in patients with SLE, even if only patients diagnosed with SLE before or at GBS onset were considered to have SLE-related GBS (9). SLE-associated polyneuropathy is preferable to describe these cases. Another major concern is that they included those cases that did not meet all criteria but where GBS or one of its synonyms was regarded as the preferred diagnosis (9). This may lead to an overestimation of GBS attributable to SLE.

Implementation of the CARE (CAse REport) guidelines is believed to improve the completeness and transparency of case reports published in the literature (14). A large majority of the included case reports (9), however, failed to adhere to the CARE (CAse REport) guidelines. Regarding the diagnosis of GBS, CSF examination has the most diagnostic value for GBS as well as CIDP. CIDP is an autoimmune disease characterized by neurological symptoms and progressive weakness, paresthesia, and sensory dysfunction and is associated with SLE (15). Although the authors stated that they ruled out cases of acute-onset CIDP (A-CIDP) (9), this is nearly impossible. Normal CSF protein levels within 1 week of onset and albumino-cytologic dissociation from the second week support a diagnosis of GBS, while a persistently elevated level of CSF may point to a diagnosis of CIDP that is defined by a subsequent chronic course (16). Serial lumbar puncture and CSF examination are rarely performed in clinical practice. GBS has occasionally been reported in immunocompromised patients. A large cohort study showed that GBS is less frequent in SLE, accounting for 1.1% of all cases with PNS involvement (16). Even in these cases, the so-called pure GBS, A-CIDP, GBS associated with the use of suppressants and an immunocompromised state cannot be differentiated from each other.

A-CIDP should be considered when a patient with possible GBS deteriorates again 8 weeks after onset or when deterioration occurs three times or more (17). Responses to different therapies may help to distinguish GBS from CIDP or SLE-associated polyneuropathy. A good response to steroids or immunosuppressive agents supports a diagnosis of CIDP or SLE-associated polyneuropathy, whereas a better response to IVIg supports GBS. Sural nerve biopsy and serial electrophysiological examinations, in some circumstances, are necessary to differentiate different polyneuropathies and make an accurate diagnosis.

Taken together, GBS can be viewed as a subgroup of polyneuropathy. GBS is not related to chronic autoimmune diseases, including SLE. Peripheral nerve involvement or polyneuropathy is common in patients with SLE as a complication of the disease. Incidental GBS does occur when researchers fail to adhere to the CARE guidelines, and the diagnostic criteria of GBS may lead to an overestimation of GBS in SLE patients. We propose that GBS should be meticulously diagnosed in patients with SLE.

## Author contributions

LJ: Writing—original draft. FN: Writing—original draft. H-LZ: Conceptualization, Supervision, Writing—review & editing.
